# Cyr61 Promotes Oral Squamous Cell Carcinoma Cell Motility via an Integrin αvβ3/αvβ5-PLC/PKC/c-Src-AP-1-ICAM-1 Signaling Axis

**DOI:** 10.7150/ijms.133127

**Published:** 2026-07-13

**Authors:** Kuan-Chou Lin, Pei-Wen Peng, Tsung-Ming Chang, Ying-Sui Sun, Chih-Hsin Tang, Ju-Fang Liu, Chia-Jung Lee

**Affiliations:** 1 School of Dentistry, College of Oral Medicine, Taipei Medical University, Taipei 110301, Taiwan.; 2 Department of Oral and Maxillofacial Surgery, Wan Fang Hospital, Taipei Medical University, Taipei 116827, Taiwan.; 3 School of Dental Technology, College of Oral Medicine, Taipei Medical University, Taipei City 110301, Taiwan.; 4 Department of Pharmacology, School of Medicine, China Medical University, Taichung 404802, Taiwan.; 5 Department of Medical Laboratory Science and Biotechnology, College of Medical and Health Science, Asia University, Taichung 413305, Taiwan.; 6 Chinese Medicine Research Center, China Medical University, Taichung 404802, Taiwan.; 7 Translational Medicine Center, Shin-Kong Wu Ho-Su Memorial Hospital, Taipei City 111045, Taiwan.; 8 School of Oral Hygiene, College of Oral Medicine, Taipei Medical University, Taipei City 110301, Taiwan.; 9 Department of Otolaryngology Head and Neck Surgery, Shin-Kong Wu Ho-Su Memorial Hospital, Taipei City 111045, Taiwan.

**Keywords:** oral squamous cell carcinoma (OSCC), Cyr61/CCN1, integrin αvβ3/αvβ5, ICAM-1, AP-1, migration

## Abstract

Oral squamous cell carcinoma (OSCC) frequently metastasizes, leading to poor patient outcomes. Cysteine-rich angiogenic inducer 61 (Cyr61/CCN1) has been implicated in cancer progression; however, the downstream mechanism driving OSCC motility remains incompletely defined. Cyr61 expression was elevated in OSCC and associated with advanced clinicopathological features. In OSCC cell lines, recombinant Cyr61 enhanced wound closure and Transwell migration and increased intercellular adhesion molecule-1 (ICAM-1) expression at both the mRNA and protein levels. ICAM-1 silencing significantly attenuated Cyr61-induced cell motility, indicating that ICAM-1 is an important downstream effector. Mechanistically, Cyr61 signaling was initiated through integrin αvβ3 and αvβ5, as neutralizing antibodies and siRNAs targeting these integrins suppressed Cyr61-induced migration and ICAM-1 expression. Cyr61 also induced phosphorylation of PLC, PKC, and c-Src, and pharmacological or siRNA-mediated inhibition of these kinases attenuated ICAM-1 upregulation and cell migration. Moreover, Cyr61 enhanced AP-1 activity via c-Jun phosphorylation, increased c-Jun occupancy at the ICAM-1 promoter, and inhibition of AP-1 signaling diminished Cyr61-driven ICAM-1 expression and motility. Together, these data define an integrin αvβ3/αvβ5-PLC-PKC-c-Src-AP-1 axis that transcriptionally upregulates ICAM-1, promoting OSCC migration and providing a mechanistic basis for targeting Cyr61-driven migratory programs in OSCC.

## Introduction

Oral squamous cell carcinoma (OSCC) is one of the most common malignancies worldwide and remains a major health burden 1. Although advances in diagnostic approaches have facilitated earlier detection, the 5-year survival rate of patients with OSCC remains unsatisfactory, largely because of aggressive local invasion and frequent metastasis 2, 3. Among these, cervical lymph-node metastasis is a major determinant of tumor-microenvironment interactions, in which cell adhesion molecules have essential roles in regulating tumor cell dissemination 4.

Intercellular adhesion molecule-1 (ICAM-1) is a transmembrane glycoprotein that mediates cell-cell interactions and modulates cellular adhesion and motility. Under physiological conditions, ICAM-1 is expressed at low basal levels, but its expression can be markedly induced by inflammatory mediators and other microenvironmental stimuli 5, 6. Aberrant ICAM-1 expression contributes to cancer progression by facilitating tumor cell adhesion, migration, and invasion, thereby promoting metastatic spread 7. Although integrin-dependent and AP-1-related signaling pathways have been implicated in motility-associated programs in OSCC, the upstream extracellular regulators that connect these pathways to ICAM-1 transcriptional activation remain incompletely defined.

The cellular communication network (CCN) family of matricellular proteins orchestrates cell-cell and cell-matrix interactions and has been increasingly implicated in carcinogenesis and metastasis 8-10. Cysteine-rich angiogenic inducer 61 (Cyr61/CCN1) is a CCN member whose expression has been associated with tumor development and progression in multiple cancer types, although its reported roles appear context dependent 11-19. Cyr61 exerts many of its effects primarily through binding to cell-surface integrins (ITG), including αvβ3 and αvβ5, thereby modulating downstream signaling pathways that govern adhesion, motility, and survival 20-23. Integrins αvβ3 and αvβ5 have been linked to malignant phenotypes in diverse cancers; nevertheless, how Cyr61-integrin signaling influences the metastatic potential of OSCC cells remains unclear 24-26.

As a matricellular CCN family protein, Cyr61 is a plausible upstream regulator of motility-related signaling in OSCC. Therefore, in the present study, we investigated whether Cyr61 promotes OSCC cell motility through integrin-dependent signaling linked to ICAM-1 upregulation, and sought to define the upstream signaling cascade connecting Cyr61 to ICAM-1 regulation. Clarifying these issues may provide mechanistic insight into OSCC metastasis and identify potential therapeutic targets for intervention.

## Materials and Methods

### Materials

U73122 (PLC inhibitor; U6756; Sigma-Aldrich, St. Louis, MO, USA), GF109203X (PKC inhibitor; ab144264; Abcam, Cambridge, UK), PP2 (Src family kinase inhibitor; SI-P0042; Sigma-Aldrich, St. Louis, MO, USA), curcumin (AP-1/c-Jun signaling inhibitor; C7727; Sigma-Aldrich, St. Louis, MO, USA), and tanshinone IIA (AP-1 signaling inhibitor; T4952; Sigma-Aldrich, St. Louis, MO, USA) were prepared according to the manufacturers' instructions. U73122, GF109203X, and PP2 were dissolved in dimethyl sulfoxide (DMSO) to prepare stock solutions at 10 mM, 20 mM, and 50 mM, respectively, and were diluted to the indicated working concentrations before use. Neutralizing antibodies against integrin αvβ3 (MAB1976) and integrin αvβ5 (MAB2019Z), as well as an anti-Cyr61 antibody (ABC102), were purchased from Merck Millipore (Burlington, MA, USA). Recombinant human Cyr61 protein was purchased from PeproTech (Catalog #120-25; Thermo Fisher Scientific, Waltham, MA, USA) and reconstituted in sterile distilled water before use. Primary antibodies against ICAM-1 (GTX100450; 1:1,000) were purchased from GeneTex (Hsinchu, Taiwan). Antibodies against phospho-PLCβ (2481S; 1:1,000), PLCβ (14247S; 1:1,000), phospho-PKC (9375S; 1:1,000), phospho-Src (5473S; 1:1,000), Src (2109S; 1:1,000), phospho-c-Jun (2361S; 1:1,000), and c-Jun (9165S; 1:1,000) were purchased from Cell Signaling Technology (Danvers, MA, USA). PKC (SC-17769; 1:1,000) was obtained from Santa Cruz Biotechnology (Dallas, TX, USA). Horseradish peroxidase-conjugated secondary antibodies and β-actin antibody (A5441; 1:10,000; Sigma-Aldrich, St. Louis, MO, USA) were used as indicated.

### Cell lines

Human OSCC cell lines SCC4, SCC25, and SAS were purchased from the Bioresource Collection and Research Center (BCRC, Hsinchu, Taiwan). SCC4 and SCC25 cells were cultured in DMEM supplemented with 10% heat-inactivated fetal bovine serum (FBS), 100 U/mL penicillin, and 0.1 mg/mL streptomycin. SAS cells were cultured in DMEM/Ham's F12 (1:1) supplemented with 10% heat-inactivated FBS, 100 U/mL penicillin, and 0.1 mg/mL streptomycin. All cell lines were maintained at 37°C in a humidified incubator with 5% CO_2_.

### Immunohistochemistry and tissue microarrays

An oral cavity squamous cell carcinoma tissue microarray (OR208; BioMax, Derwood, MD, USA), including 9 normal oral tissues and OSCC specimens (stage I, n = 25; stage II, n = 18; stage III, n = 6; stage IV, n = 11), was used for immunohistochemical analysis. After deparaffinization and rehydration, sections were treated with 3% hydrogen peroxide to block endogenous peroxidase activity. Antigen retrieval was performed as described previously, followed by blocking with 3% BSA in PBS. Sections were incubated with anti-Cyr61 antibody (1:100) at 4 °C overnight and subsequently with a biotin-labeled secondary antibody (1:100). Immunoreactivity was visualized using an ABC kit (Vector Laboratories, Burlingame, CA, USA) with DAB as the chromogen, followed by counterstaining with Delafield's hematoxylin. Images were captured using a Nikon ECLIPSE Ti microscope under identical imaging conditions. Negative controls were prepared by omitting the primary antibody or replacing it with non-immune IgG under otherwise identical staining conditions; no specific signal was detected in the negative controls. A total of 60 evaluable tissue cores were included in the final analysis, whereas cores with insufficient tissue, severe tissue loss, folding artifacts, or poor staining quality were excluded. Cyr61 immunoreactivity was quantified using ImageJ software by measuring the DAB-positive staining area relative to the total tissue area in each core. The staining result was expressed as Cyr61-positive staining area (%) = DAB-positive stained area / total tissue area × 100. Quantified values were grouped according to clinical stage and subjected to statistical analysis.

### Online database analysis

Cyr61 expression and clinical information were retrieved from TCGA and analyzed using UALCAN 27. Kaplan-Meier overall survival curves were generated for the TCGA oral cavity/OSCC subset by stratifying patients according to the median Cyr61 expression level. Univariate and multivariate Cox proportional hazards analyses were performed, with the multivariate model adjusted for clinical stage, age, and sex. Cyr61 expression and survival were further assessed using the R2 Genomics Analysis and Visualization Platform in TCGA-HNSCC datasets. In addition, Cyr61 expression in OSCC was examined in GEO datasets, including GSE30784 (OSCC tissues and adjacent noncancerous tissues) 27, 28.

### Cell movement assay

For the wound-healing assay, OSCC cells were seeded in 12-well plates at a density of 5 × 10^4^ cells/well and cultured until a near-confluent monolayer formed. After the indicated pretreatments, a linear wound was created across the cell monolayer using a sterile pipette tip. Detached cells were removed by washing with PBS, and fresh medium containing the indicated treatments was added. Wound closure was monitored using a Nikon ECLIPSE Ti microscope equipped with NIS-Elements AR software (version 5.02.01). Images were obtained at 0 and 24 h, and the wound area was quantified using ImageJ software (1.53a). Cell movement was expressed relative to the control group.

### Cell migration assay

Pretreated OSCC cells (3 × 10^4^) were seeded in the upper chambers of Corning Transwell inserts with a pore size of 8.0 μm in a 24-well plate to determine migration capacity. Cell culture media (300 μL containing 1% FBS) were added to the lower chamber. After incubation under 5% CO_2_ at 37 °C for 24 h, each transwell chamber was removed from the plate for the removal of non-migrating cells from the upper chamber using cotton swabs. The cells that migrated to the lower surface were stained with 0.05% crystal violet in 20% methanol for fixation. ImageJ (1.53a) software was used to quantify the number of cells. Untreated cells were used as controls.

### Antibody-blocking experiments

For the antibody-blocking experiments, OSCC cells were preincubated with control IgG or neutralizing antibodies against integrin αvβ3 or integrin αvβ5 at 100 pg/mL for 30 min before stimulation with recombinant human Cyr61 protein. To neutralize endogenous Cyr61, OSCC cells were incubated with control IgG or a neutralizing anti-Cyr61 antibody at 100 pg/mL for 24 h without recombinant Cyr61 stimulation. After the indicated antibody treatments, cell movement, migration, and ICAM-1 expression were analyzed.

### RNA extraction and quantitative real-time polymerase chain reaction

Total RNA was extracted using TRIzol reagent (Sigma-Aldrich) and reverse-transcribed (1 µg RNA) into cDNA using an RT kit (PCR Biosystems) according to the manufacturer's instructions. qPCR was performed using KAPA SYBR FAST qPCR Master Mix on a CFX Connect system (Bio-Rad). Relative expression was calculated using the 2^-ΔΔCt^ method with GAPDH as an internal control. Primer sequences were human ICAM-1: forward, 5'- ACCATCTACAGCTTTCCG-3', reverse, 5'- TCACACTTCACTGTCACC-3'; human GAPDH: forward: 5'- ACAGTTGCCATGTAGACC-3', reverse, 5'- TTGAGCACAGGGTACTTTA-3'.

### ELISA for detecting human Cyr61 protein

Secreted Cyr61 protein levels were quantified using a Human Cyr61 ELISA Kit (ab238267; Abcam, Cambridge, MA, USA) according to the manufacturer's instructions. Cell culture supernatants were collected and clarified by centrifugation prior to analysis. Briefly, standards and samples were added to the antibody-coated microplate, followed by incubation with the antibody cocktail provided in the kit. After washing, the signal was developed using TMB substrate and measured at 450 nm using a microplate reader. Cyr61 concentrations were calculated from the standard curve and expressed as indicated.

### Protein extraction and immunoblotting analysis

After washing OSCC cells using PBS, the cell lysate was collected using RIPA lysis buffer containing protease inhibitors. Cells were scraped and centrifuged at 15,000 × g at 4 °C for 30 min to obtain whole cell extract. Proteins were separated via 8-12% SDS-PAGE and transferred to PVDF membranes. The PVDF membranes were then blocked with 5% skim milk at room temperature for 1 h and then washed with TBST three times prior to incubation with primary antibodies (1:1,000 dilution) on a shaker at 4°C overnight. Following incubation with primary antibodies, the PVDF membranes were washed using TBST three times and incubated with horseradish peroxidase (HRP)-conjugated secondary antibodies (1:10,000 dilution) at room temperature for 1 h. Blots were visualized using ECL SelectTM Western Blotting Detection Reagent (Cytiva; RPN2235) and exposed using a UVP ChemiDot-It 815 Imager BioImaging System.

### Small-interfering RNA transfection

OSCC cells were seeded in 6-well plates and transfected with siRNAs targeting ICAM-1, ITGAV, ITGB3, ITGB5, PLCB3, PKC, SRC, and JUN using Lipofectamine 3000 (Invitrogen, Carlsbad, CA, USA) according to the manufacturer's instructions. Briefly, siRNA was used at a final concentration of 100 nM per well and diluted in 25 µL serum-free medium, while 1 µL Lipofectamine 3000 Reagent was diluted separately in 25 µL serum-free medium. The two solutions were then combined and incubated for 10 min at room temperature before being added to each well. On the following day, the medium was replaced with complete medium, and cells were treated with recombinant Cyr61 (30 ng/mL) as indicated before subsequent experiments. siRNA sequences were: ICAM-1 5′-GGAACAACCGGAAGGUGUA-3′; ITGAV 5′-GAAUAUCGGUUGGAUUAUA-3′; ITGB3 5′-GCAAUGUCCUCCAGCUCAU-3′; ITGB5 5′-GCUAUGAAAUGGCUUCAAA-3′; PLCB3 5′-GAUGUACCGCCAGGCACUA-3′; PKC 5′-GAGUUUCGGAGCUGAUGAA-3′; SRC 5′-CAGUUGUAUGCUGUGGUUU-3′; JUN 5′-GGAUCAAGGCGGAGAGGAA-3′.

### Activator protein 1 (AP-1) luciferase activity

AP-1-responsive promoter elements were subcloned into the pGL4.44 luciferase reporter vector (Promega, Madison, WI, USA). SCC4 and SAS cells were seeded in 12-well plates and transfected with the AP-1 luciferase reporter construct using Lipofectamine 3000 according to the manufacturer's instructions. Briefly, 500 ng plasmid DNA and 1 µL P3000 Reagent were diluted in 25 µL serum-free medium, while 0.75 µL Lipofectamine 3000 Reagent was diluted separately in 25 µL serum-free medium. The two solutions were then combined and incubated for 10 min at room temperature before being added to each well. On the following day, cells were pretreated with the indicated inhibitors for 1 h and then stimulated with recombinant Cyr61. Luciferase activity was measured using the Promega luciferase reporter assay system according to the manufacturer's instructions and was normalized to total protein concentration.

### Chromatin immunoprecipitation (ChIP) assay

Cells were pretreated with the indicated inhibitors for 1 h and then stimulated with recombinant Cyr61 as described in the figure legends. Following treatment, cells were cross-linked with 4% formaldehyde for 15 min at room temperature and the reaction was quenched by adding glycine. Cells were then washed with cold PBS and lysed, and chromatin was sheared by sonication to generate DNA fragments of approximately 200-500 bp. The sheared chromatin was immunoprecipitated with an anti-c-Jun antibody. After washing, protein-DNA complexes were eluted and cross-links were reversed, followed by DNA purification. The recovered DNA was subjected to PCR analysis to evaluate c-Jun binding to the ICAM-1 promoter region. PCR products were resolved on agarose gels and visualized under UV illumination. The primer sequences used for amplification of the ICAM-1 promoter (c-Jun binding region) were as follows: forward 5′-AGACCTTAGCGCGGTGTAGA-3′ and reverse 5′-AGTAGCAGAGGAGCTCAGCG-3′.

### Statistical analysis

All experiments were performed at least four independent times. Data are presented as mean ± standard deviation (SD). Statistical analyses were performed using GraphPad Prism software. For comparisons among multiple groups, one-way ANOVA was applied followed by post hoc multiple-comparison tests as implemented in Prism: Tukey's test was used when comparing all groups with each other, whereas Dunnett's test was used when comparisons were limited to the control group. A *p* value < 0.05 was considered statistically significant.

## Results

### Cyr61 is upregulated in OSCC and associated with advanced clinicopathological features

**Figure 1:**
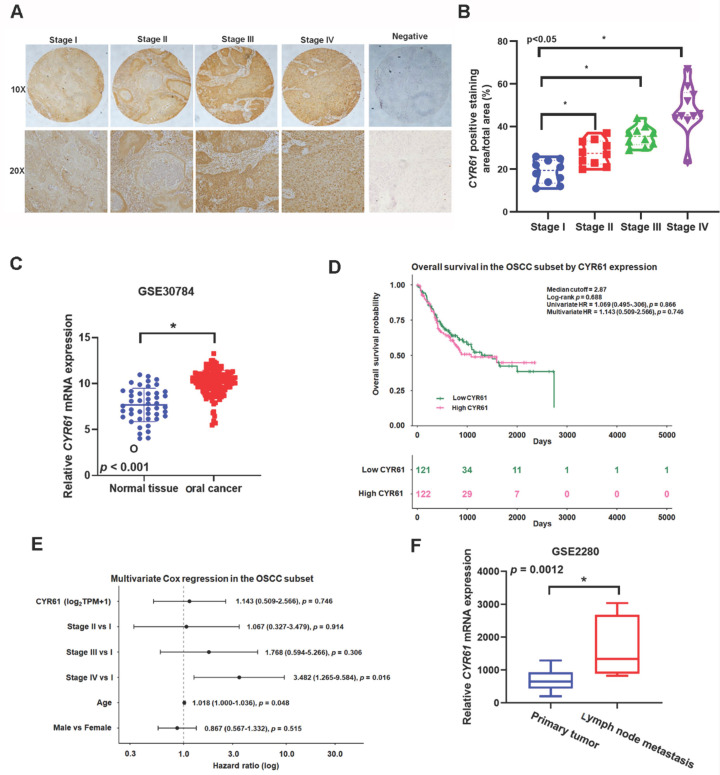
** Cyr61 expression is elevated in OSCC and associated with advanced clinicopathological features.** (A) Representative immunohistochemical staining of Cyr61 in OSCC tissue specimens from stages I-IV, together with the negative control. (B) Quantification of Cyr61-positive staining area in OSCC specimens according to clinical stage. (C) Cyr61 mRNA expression in normal oral mucosa and OSCC tissues from the GEO dataset GSE30784. (D) Kaplan-Meier overall survival analysis of the TCGA oral cavity/OSCC subset stratified by median Cyr61 expression, with annotation of the log-rank *p* value and the corresponding univariate and multivariate Cox regression results. (E) Forest plot of multivariate Cox proportional hazards analysis in the TCGA oral cavity/OSCC subset, showing hazard ratios for Cyr61 expression, clinical stage, age, and sex. (F) Cyr61 mRNA expression in metastatic lymph node samples and primary tumor samples from OSCC patients in the GEO dataset GSE2280. *p < 0.05 as indicated.

To assess the clinical relevance of Cyr61 in OSCC, we examined Cyr61 expression in tissue specimens and public transcriptomic datasets and further evaluated its association with clinicopathological features and survival. Immunohistochemical analysis of the tissue microarray showed stronger Cyr61 staining across advanced OSCC stages, and Cyr61 expression was significantly associated with clinical stage (Figure 1A, B). Consistent with this observation, analysis of the GSE30784 dataset showed that Cyr61 mRNA expression was significantly higher in OSCC tissues than in normal oral mucosa (Figure 1C). We next re-analyzed overall survival in the TCGA oral cavity/OSCC subset. Kaplan-Meier analysis stratified by the median Cyr61 expression level showed no significant difference in overall survival between the high- and low-expression groups (log-rank *p* = 0.688) (Figure 1D). In univariate Cox analysis, Cyr61 expression was not significantly associated with overall survival (HR = 1.069, 95% CI = 0.495-2.306, *p* = 0.866). Likewise, in multivariate Cox regression adjusting for stage, age, and sex, Cyr61 remained non-significant (HR = 1.143, 95% CI = 0.509-2.566, *p* = 0.746), whereas Stage IV disease and older age were independently associated with worse overall survival (Figure 1E). In addition, analysis of the GSE2280 dataset showed increased Cyr61 mRNA expression in metastatic lymph node samples compared with primary tumor samples from OSCC patients (Figure 1F). Collectively, these findings indicate that Cyr61 is upregulated in OSCC and associated with advanced clinicopathological features; however, it was not identified as an independent prognostic factor for overall survival in the TCGA OSCC subset.

### Cyr61 promotes OSCC cell motility by inducing ICAM-1 expression

**Figure 2:**
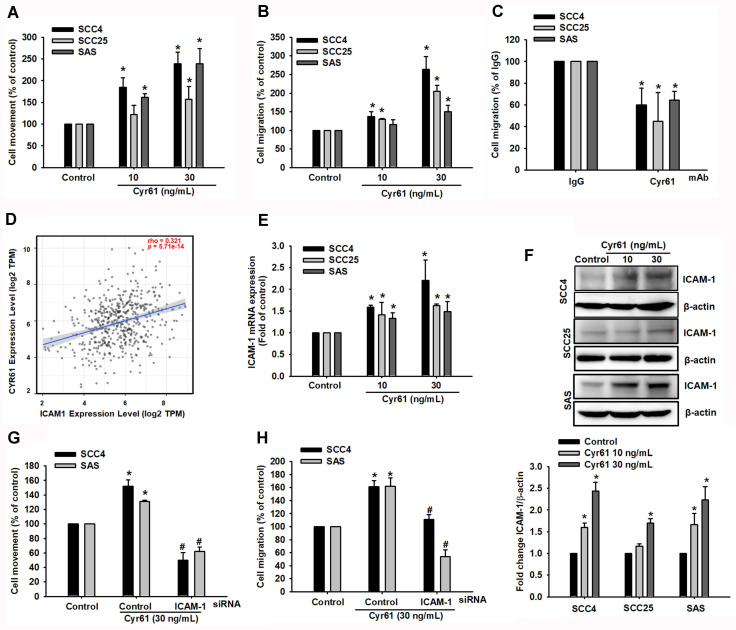
** Cyr61 enhances OSCC motility and migration through upregulation of ICAM-1 expression.** (A, B) OSCC cells were treated with recombinant Cyr61 at the indicated concentrations for 24 h, and (A) cell movement (wound-healing assay) and (B) cell migration (Transwell assay) were assessed (n = 4). (C) Quantification of cell migration in OSCC cells preincubated with control IgG or anti-Cyr61 antibody (100 pg/mL) for 24 h (n = 4). (D) Correlation analysis between Cyr61 and ICAM-1 expression in the TCGA-HNSC cohort using TIMER2.0. Spearman correlation. (E) ICAM-1 mRNA expression following Cyr61 treatment (10-30 ng/mL) (n = 4). (F) ICAM-1 protein expression following Cyr61 treatment at the indicated concentrations (n = 4). (G, H) Quantification of (G) cell movement and (H) cell migration following control and ICAM-1 siRNA transfection and Cyr61 stimulation (30 ng/mL) (n = 4). Data are presented as mean ± SD of four independent experiments. *p < 0.05 compared with the control group; #*p* < 0.05 compared with the Cyr61-treated group.

To test whether Cyr61 directly enhances OSCC cell motility, we treated OSCC cell lines with recombinant Cyr61 and assessed wound closure and Transwell migration. ELISA analysis of conditioned media revealed differential endogenous Cyr61 secretion among OSCC cell lines, with SAS cells releasing higher Cyr61 levels than SCC25 cells (Figure S1C). Using SCC4, SCC25, and SAS cells, wound-healing and Transwell assays demonstrated that recombinant Cyr61 stimulation increased wound closure and cell migration (Figure 2A-B). A dose-response analysis further showed that Cyr61 increased SAS cell migration in a concentration-dependent manner, with significant enhancement observed from 30 ng/mL (Figure S1D). Recombinant Cyr61 did not significantly affect the proliferation of SCC4, SCC25, or SAS cells under the experimental conditions (Figure S1A), indicating that the enhanced wound closure was not attributable to increased cell growth. Consistent with the functional role of Cyr61, neutralization of endogenous Cyr61 expressed by cancer cells using a Cyr61-neutralizing monoclonal antibody (100 pg/mL) significantly suppressed OSCC cell migration (Figure 2C). These findings suggest that cancer cell-derived Cyr61 contributes to the migratory phenotype. Because cell adhesion molecules are central to metastatic dissemination, we next examined ICAM-1 as a potential downstream mediator 29, 30. TIMER2.0 analysis of the TCGA-HNSC cohort showed a positive correlation between Cyr61 and ICAM-1 expression (*p* < 0.0001, rho = 0.321) (Figure 2D) 31. Experimentally, stimulation with recombinant Cyr61 increased ICAM-1 protein and mRNA levels in a dose-dependent manner in SCC4, SCC25 and SAS cells (Figure 2E-F). Consistently, Cyr61 induced ICAM-1 mRNA expression in SAS cells in a dose-dependent manner, with significant induction starting at 30 ng/mL (Figure S1E). At the protein level, Cyr61 increased ICAM-1 expression in SAS cells, with a significant elevation observed from 50 ng/mL (Figure S1F). To test whether ICAM-1 is required for Cyr61-induced motility, we silenced ICAM-1 using siRNA. ICAM-1 knockdown (100 nM) markedly attenuated Cyr61-induced wound closure and Transwell migration (Figure 2G-H). Moreover, knockdown of Cyr61 in SAS cells reduced ICAM-1 protein expression (Figure S1H), further supporting Cyr61 as an upstream regulator of ICAM-1 in OSCC. These results support ICAM-1 as a necessary downstream effector mediating Cyr61-driven motility in OSCC cells.

### Cyr61 promotes cell migration and ICAM-1 upregulation through integrin αvβ3 and αvβ5 in OSCC cells

**Figure 3:**
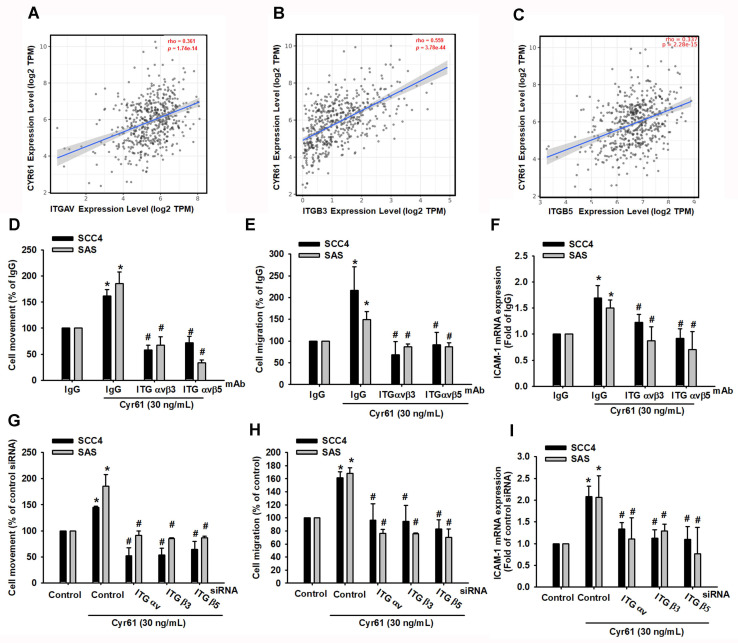
** Cyr61 promotes OSCC cell motility and ICAM-1 expression through integrin αvβ3 and αvβ5.** (A-C) Correlation analysis of Cyr61 with (A) ITGAV, (B) ITGB3, and (C) ITGB5 expression in TCGA-HNSC cohort using TIMER2.0. Spearman correlation. (D-F) Quantification of (D) cell movement, (E) cell migration, and (F) ICAM-1 mRNA expression in OSCC cells preincubated with control IgG, a neutralizing antibody against integrin αvβ3, or a neutralizing antibody against integrin αvβ5 (100 ng/mL) for 30 min before stimulation with recombinant human Cyr61 protein (n = 4). (G-I) Quantification of (G) cell movement, (H) cell migration, and (I) ICAM-1 mRNA expression following siRNA transfection targeting ITGAV, ITGB3, or ITGB5 and Cyr61 stimulation (30 ng/mL) (n = 4). Data are presented as mean ± SD of four independent experiments. *p < 0.05 compared with the control or IgG group; *#p* < 0. 05 compared with the Cyr61-treated group.

Cyr61 is known to regulate cellular behavior through binding to specific cell-surface integrins 20, 21, 32. We therefore investigated whether integrin αvβ3 and αvβ5 participate in Cyr61-mediated responses in OSCC. First, TIMER2.0 analysis of TCGA-HNSC data showed that Cyr61 expression positively correlates with ITGAV, ITGB3, and ITGB5 expression (*p* < 0.0001; rho = 0.361, 0.559, and 0.337, respectively) (Figure 3A-C). We next tested functional involvement in vitro. Pretreatment with neutralizing antibodies against integrin αvβ3 or αvβ5 significantly suppressed Cyr61-induced wound closure, Transwell migration, and ICAM-1 expression (Figure 3D-F). These findings were independently confirmed by gene silencing: siRNAs targeting ITGAV, ITGB3, or ITGB5 significantly reduced Cyr61-induced motility and ICAM-1 expression (Figure 3G-I). Together, these data demonstrate that integrin αvβ3 and αvβ5 act upstream of Cyr61-induced ICAM-1 upregulation and motility in OSCC cells.

### PLC/PKC/c-Src activation is required for Cyr61-induced ICAM-1 expression and OSCC motility

**Figure 4:**
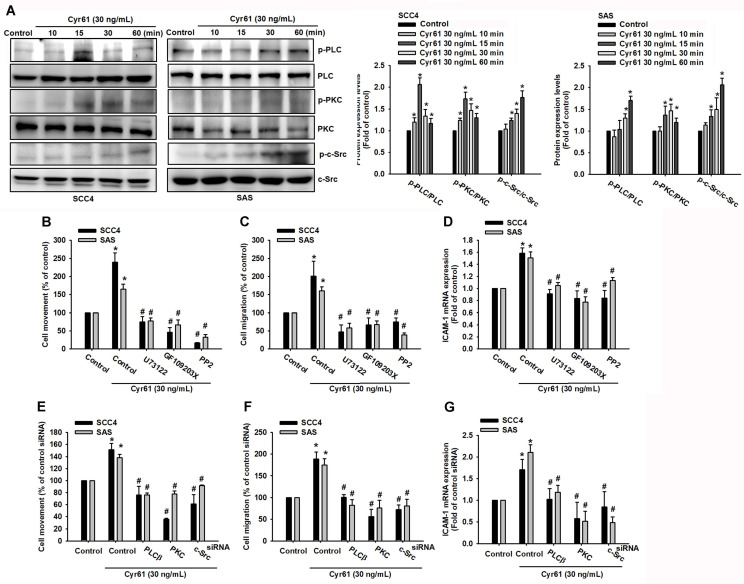
** PLC/PKC/c-Src signaling is required for Cyr61-induced ICAM-1 expression and OSCC migration.** (A) Phosphorylation of PLC, PKC, and c-Src in SCC4 and SAS cells following Cyr61 stimulation (n = 4). (B-D) Quantification of (B) cell movement, (C) cell migration, and (D) ICAM-1 mRNA expression in cells pretreated with inhibitors of PLC, PKC, or c-Src (U73122, 1 µM; GF109203X, 5 µM; or PP2, 5 µM), followed by Cyr61 stimulation (30 ng/mL) (n = 4). (E-G) Quantification of (E) cell movement, (F) cell migration, and (G) ICAM-1 mRNA expression following siRNA-mediated knockdown of PLC, PKC, or c-Src and Cyr61 stimulation (30 ng/mL) (n = 4). Data are presented as mean ± SD of four independent experiments. *p < 0.05 compared with the control group; #*p* < 0.05 compared with the Cyr61-treated group.

Integrin engagement commonly activates various kinase cascades, including phospholipase C (PLC), protein kinase C (PKC), and proto-oncogene tyrosine-protein kinase Src (c-Src), that regulate motility-related gene programs 33. We therefore examined whether PLC, PKC, and c-Src signaling contribute to Cyr61-driven OSCC migration and ICAM-1 induction. Stimulation with Cyr61 rapidly increased phosphorylation of PLC, PKC, and c-Src within 15 minutes (Figure 4A), indicating activation of these pathways. To determine functional relevance, we pharmacologically inhibited each kinase prior to Cyr61 stimulation. Inhibition of PLC (U73122, 1 µM), PKC (GF109203X, 5 µM), or c-Src (PP2, 5 µM) significantly reduced Cyr61-induced cell movement, migration, and ICAM-1 mRNA upregulation (Figure 4B-D). At the doses used, U73122, GF109203X, and PP2 did not significantly alter OSCC cell proliferation (Figure S1B), supporting that their inhibitory effects on Cyr61-induced motility and ICAM-1 expression were not due to cytotoxicity. Consistently, siRNA-mediated knockdown of PLC, PKC, or c-Src similarly attenuated Cyr61-induced motility and ICAM-1 expression (Figure 4E-G). These results indicate that PLC, PKC, and c-Src signaling are critical components of the Cyr61-integrin pathway leading to ICAM-1 induction and enhanced OSCC cell motility.

### AP-1/c-Jun transcriptionally drives Cyr61-induced ICAM-1 expression and OSCC motility

**Figure 5:**
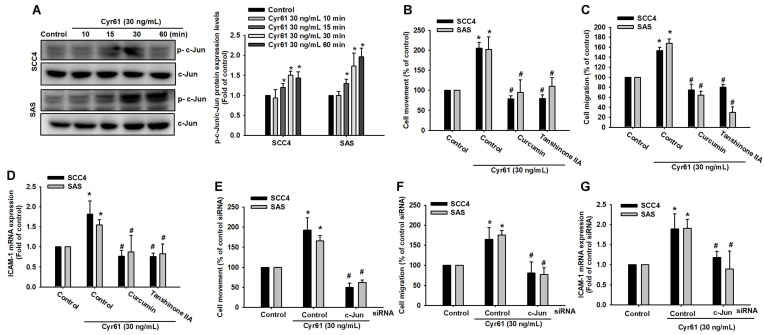
** AP-1/c-Jun signaling mediates Cyr61-induced ICAM-1 expression and OSCC migration.** (A) Phosphorylation of c-Jun following Cyr61 stimulation (n = 4). (B-D) Quantification of (B) cell movement, (C) cell migration, and (D) ICAM-1 mRNA expression in cells pretreated with curcumin (1 μM) or tanshinone IIA (5 μM) for 1 h before stimulation with Cyr61 (30 ng/mL) (n = 4). (E-G) Quantification of (E) cell movement, (F) cell migration, and (G) ICAM-1 mRNA expression following c-Jun siRNA transfection and Cyr61 stimulation (30 ng/mL) (n = 4). Data are presented as mean ± SD of four independent experiments. *p < 0.05 compared with the control group; #*p* < 0.05 compared with the Cyr61-treated group.

**Figure 6:**
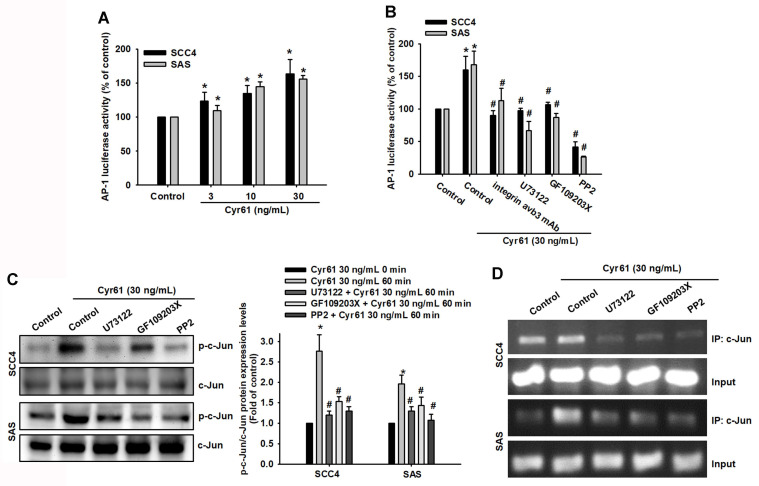
** Cyr61 activates AP-1 and enhances c-Jun binding to the ICAM-1 promoter in OSCC cells.** (A) AP-1 luciferase activity following Cyr61 stimulation (3-30 ng/mL) (n = 4). (B) AP-1 luciferase activity in cells pretreated with an anti-integrin αvβ3 antibody or with inhibitors of PLC, PKC, or c-Src (U73122, 1 µM; GF109203X, 5 µM; or PP2, 5 µM), followed by Cyr61 stimulation (30 ng/mL) (n = 4). (C) c-Jun phosphorylation in cells pretreated with PLC, PKC, or c-Src inhibitors prior to Cyr61 stimulation (30 ng/mL) (n = 4). (D) Chromatin immunoprecipitation (ChIP) analysis showing c-Jun binding to the AP-1 recognition sequence within the ICAM-1 promoter following pretreatment with specific inhibitors and Cyr61 stimulation (30 ng/mL) (n = 4). Data are presented as mean ± SD of four independent experiments. *p < 0.05 compared with the control group; #*p* < 0.05 compared with the Cyr61-treated group.

Because AP-1 is a known transcriptional regulator of ICAM-1 34-37, we next investigated whether AP-1 mediates Cyr61-induced ICAM-1 expression and motility in OSCC cells. Cyr61 stimulation induced time-dependent phosphorylation of c-Jun (Figure 5A). Pharmacological inhibition of AP-1 signaling (curcumin and tanshinone IIA, as indicated) significantly decreased Cyr61-induced cell movement, migration, and reduced ICAM-1 mRNA expression (Figure 5B-D). Similarly, siRNA-mediated silencing of c-Jun markedly suppressed Cyr61-driven motility and ICAM-1 mRNA induction (Figure 5E-G), supporting a functional requirement for c-Jun/AP-1. We then asked whether the upstream PLC-PKC-c-Src cascade links Cyr61-integrin signaling to AP-1 activation. Cyr61 increased AP-1 reporter activity in a dose-dependent manner (Figure 6A). Importantly, pretreatment with a neutralizing antibody against integrin αvβ3, as well as pharmacological inhibition of PLC, PKC, or c-Src, significantly reduced Cyr61-induced AP-1 luciferase activity (Figure 6B), indicating that integrin αvβ3 and the downstream PLC-PKC-c-Src cascade are required for Cyr61-mediated AP-1 activation. Consistently, inhibition of PLC, PKC, or c-Src also attenuated Cyr61-induced c-Jun phosphorylation (Figure 6C). Furthermore, ChIP analysis showed that pharmacological inhibition of PLC, PKC, or c-Src diminished Cyr61-enhanced c-Jun binding to the AP-1 recognition sequence within the ICAM-1 promoter (Figure 6D). Collectively, these results support a model in which Cyr61 activates integrin αvβ3/αvβ5-dependent PLC-PKC-c-Src signaling, leading to c-Jun/AP-1 activation and transcriptional upregulation of ICAM-1, thereby promoting OSCC cell motility.

## Discussion

Metastatic dissemination, particularly cervical lymph node involvement, remains a major determinant of poor prognosis in oral squamous cell carcinoma (OSCC) 2, 3. Here, we identify ICAM-1 as a required downstream effector of Cyr61-driven motility in OSCC and define the upstream signaling cascade controlling its transcription. Cyr61 was elevated in OSCC and associated with advanced clinicopathological features. However, Cyr61 was not identified as an independent prognostic factor for overall survival in the TCGA OSCC subset. In vitro, Cyr61 enhanced wound closure and Transwell migration, and this phenotype was abolished by ICAM-1 silencing. Mechanistically, our pharmacologic, siRNA, reporter, and ChIP data support a causal pathway in which Cyr61 engages integrin αvβ3/αvβ5 to activate PLC-PKC-c-Src signaling, leading to AP-1/c-Jun-dependent transcriptional upregulation of ICAM-1. Together, these findings suggest that Cyr61 contributes to motility-related signaling in OSCC and is associated with aggressive disease features, but its value as an independent prognostic biomarker for overall survival was not supported in this cohort.

Cyr61 (CCN1) is a matricellular protein of the CCN family that regulates cell-cell and cell-matrix interactions and has been implicated in tumor progression in a context-dependent manner 8-10. Previous studies have reported that Cyr61 is frequently elevated in OSCC and can be associated with aggressive clinicopathological features and poor clinical outcomes 15. In addition, transcriptome profiling has linked Cyr61 to invasive phenotypes in oral cancer, and Cyr61 knockdown has been shown to suppress malignant behaviors, including cell growth and motility-related phenotypes, in OSCC models 38. In addition to ICAM-1 induction, Cyr61 stimulation increased the mRNA expression of multiple MMPs (MMP1/2/3/9/12/13) in SAS cells (Figure S1G), suggesting that Cyr61 may broadly activate pro-migratory and matrix-remodeling programs in OSCC. Consistent with these reports, our clinical and public dataset analyses indicate that Cyr61 is upregulated in OSCC and enriched in metastatic lymph node samples, supporting the clinical relevance of Cyr61-associated signaling programs in OSCC progression 15, 38. The conceptual novelty of the present study should be interpreted with appropriate caution. Prior studies, including our previous work, have already implicated CCN-family proteins and integrin/AP-1-related signaling in OSCC motility. Thus, the present study does not establish the first general link between integrin/AP-1 signaling and motility-associated phenotypes in OSCC. Rather, its main contribution lies in identifying Cyr61 as an upstream extracellular regulator of a defined integrin αvβ3/αvβ5-PLC/PKC/c-Src-AP-1-ICAM-1 signaling cascade that promotes motility-related behavior in OSCC.

A major finding of this study is that Cyr61-driven OSCC motility requires integrin αvβ3 and αvβ5. Cyr61 exerts biological functions through direct binding to integrins, leading to activation of intracellular signaling pathways controlling adhesion and migration 20-23. Integrins, particularly αv-containing integrins, are well-established regulators of tumor progression and represent key signaling hubs that couple extracellular matrix engagement to cytoskeletal remodeling and motile behavior 24-26. In our study, both neutralizing antibodies and siRNA-mediated knockdown of integrin αvβ3 or αvβ5 significantly reduced Cyr61-induced wound closure, Transwell migration, and ICAM-1 expression, placing integrin αvβ3/αvβ5 upstream of Cyr61-mediated responses. These results support a model in which Cyr61 initiates a pro-migratory program through αvβ3/αvβ5-dependent signaling in OSCC cells.

Downstream of integrin engagement, we identified PLC, PKC, and c-Src as essential intermediates connecting Cyr61 stimulation to ICAM-1 induction and motility. Integrin-dependent signaling commonly converges on kinase networks involving PLC/PKC and Src family kinases, which coordinate focal adhesion turnover and migration 33, 39, 40. We observed rapid phosphorylation of PLC, PKC, and c-Src following Cyr61 stimulation, and both pharmacological inhibition and siRNA-mediated knockdown of these kinases attenuated Cyr61-induced migration and ICAM-1 upregulation. These findings indicate that PLC-PKC-c-Src signaling is functionally required for transmitting Cyr61-integrin signals to downstream gene regulation in OSCC cells. Mechanistically, this framework is consistent with prior evidence that ICAM-1 induction can involve PLC/PKC-dependent Src activation and integrin-associated signaling modules that regulate motility-related outputs 41-43.

ICAM-1 emerged as a critical effector linking Cyr61 signaling to OSCC motility. ICAM-1 is a transmembrane adhesion molecule that is typically low at baseline but can be induced by inflammatory and microenvironmental stimuli, contributing to tumor cell adhesion, migration, and dissemination 5-7. Increasing evidence supports the importance of cell adhesion molecules in cancer plasticity and metastasis-related phenotypes, and recent reviews further highlight ICAM-1 as a clinically relevant mediator in cancer progression 29, 30. In OSCC, ICAM-1 expression correlates with disease progression and can facilitate interactions between cancer cells and immune/stromal components within the tumor microenvironment 5. Our data extend these observations by identifying Cyr61 as an upstream regulator that robustly induces ICAM-1 at both mRNA and protein levels and by demonstrating a functional requirement for ICAM-1 in Cyr61-driven motility/migration. Thus, ICAM-1 may act as a mechanistic bridge connecting extracellular Cyr61 signaling to an adhesive, pro-migratory phenotype in OSCC.

At the transcriptional level, our findings further establish AP-1/c-Jun as a key downstream node that mediates Cyr61-induced ICAM-1 expression. AP-1 is a central transcription factor complex implicated in motility-related gene programs and has been shown to regulate ICAM-1 expression through promoter binding and transcriptional activation 34, 35. AP-1 activity can be modulated by upstream kinase cascades, and c-Jun phosphorylation is a hallmark of AP-1 activation 36, 37. In this study, Cyr61 induced time-dependent c-Jun phosphorylation, enhanced AP-1 reporter activity, and increased c-Jun occupancy at the ICAM-1 promoter. Inhibition of PLC, PKC, or c-Src suppressed AP-1 activation and reduced c-Jun binding to the ICAM-1 promoter. These data place PLC-PKC-c-Src upstream of AP-1-dependent ICAM-1 transcription in response to Cyr61-integrin αvβ3/αvβ5 signaling. These mechanistic results provide direct promoter-level evidence that strengthens causal inference beyond association and supports a coherent pathway linking Cyr61 stimulation to ICAM-1 transcription and motility.

From a translational perspective, the Cyr61-integrin-ICAM-1 axis may represent a potential anti-metastatic therapeutic vulnerability in OSCC. Cyr61 is a secreted protein, making it potentially amenable to extracellular targeting strategies, while integrins and Src-dependent signaling represent druggable nodes that have been extensively explored in oncology 20, 21, 24, 25, 32, 33. Moreover, given the emerging clinical relevance of ICAM-1 in cancer progression, ICAM-1 induction may serve as a functional biomarker of Cyr61-driven pro-migratory signaling and help stratify patients at higher risk for dissemination 29, 30. Future studies may determine whether targeting Cyr61 signaling, integrin αvβ3/αvβ5 engagement, or downstream kinases can effectively suppress Cyr61-associated migratory programs and thereby limit OSCC progression.

Several limitations of this study should be acknowledged. First, although our results define a signaling cascade and support ICAM-1 as an important downstream effector of Cyr61 in promoting OSCC cell motility in vitro, further validation using invasion assays and in vivo models would strengthen the physiological and pathological relevance of this pathway. Second, although siRNA-mediated knockdown supported the pharmacological findings, additional rescue experiments or isoform-specific genetic approaches would further strengthen pathway specificity 36, 37. Third, although Cyr61 expression was associated with advanced clinicopathological features in our clinical and public database analyses, our re-analysis of the TCGA oral cavity/OSCC subset did not support Cyr61 as an independent prognostic factor for overall survival. Accordingly, the clinical significance of Cyr61 in the present study should be interpreted cautiously, primarily in relation to tumor aggressiveness and motility-related behavior rather than as a standalone survival biomarker. Further validation in larger and independent OSCC cohorts will be important to clarify the clinicopathological relevance of Cyr61 in oral cancer. Despite these limitations, our study identifies a Cyr61-integrin αvβ3/αvβ5-PLC/PKC/c-Src-AP-1 signaling axis that transcriptionally upregulates ICAM-1 and promotes OSCC motility, providing mechanistic insight into motility-related signaling in OSCC.

## Conclusion

This study provides mechanistic insight into Cyr61-driven motility-related signaling in OSCC. We demonstrate that Cyr61 promotes OSCC cell motility by inducing ICAM-1 expression through integrin αvβ3/αvβ5 engagement and activation of the PLC-PKC-c-Src-AP-1 signaling cascade. These findings identify a Cyr61-ICAM-1 regulatory axis that may contribute to aggressive tumor behavior in OSCC and provide a basis for future studies targeting Cyr61-driven motility-related pathways.

## Supplementary Material

Supplementary figure.

## Data Availability

The public datasets analyzed in this study are available from TCGA and GEO (GSE30784 and GSE2280). These datasets were accessed and analyzed using UALCAN, TIMER2.0, and the R2 Genomics Analysis and Visualization Platform. The experimental data generated and analyzed during the current study are available from the corresponding author upon reasonable request.
