# Supplementary material for: Cyr61 Promotes Oral Squamous Cell Carcinoma Cell Motility via an Integrin αvβ3/αvβ5-PLC/PKC/c-Src-AP-1-ICAM-1 Signaling Axis
Source: Int J Med Sci. 2026 Jul 13;23(8):2657–69. doi: 10.7150/ijms.133127 (PMC13411012; doi:10.7150/ijms.133127)
Supplement: Supplementary file 1 — Supplementary figure. [file ijmsv23p2657s1.pdf]

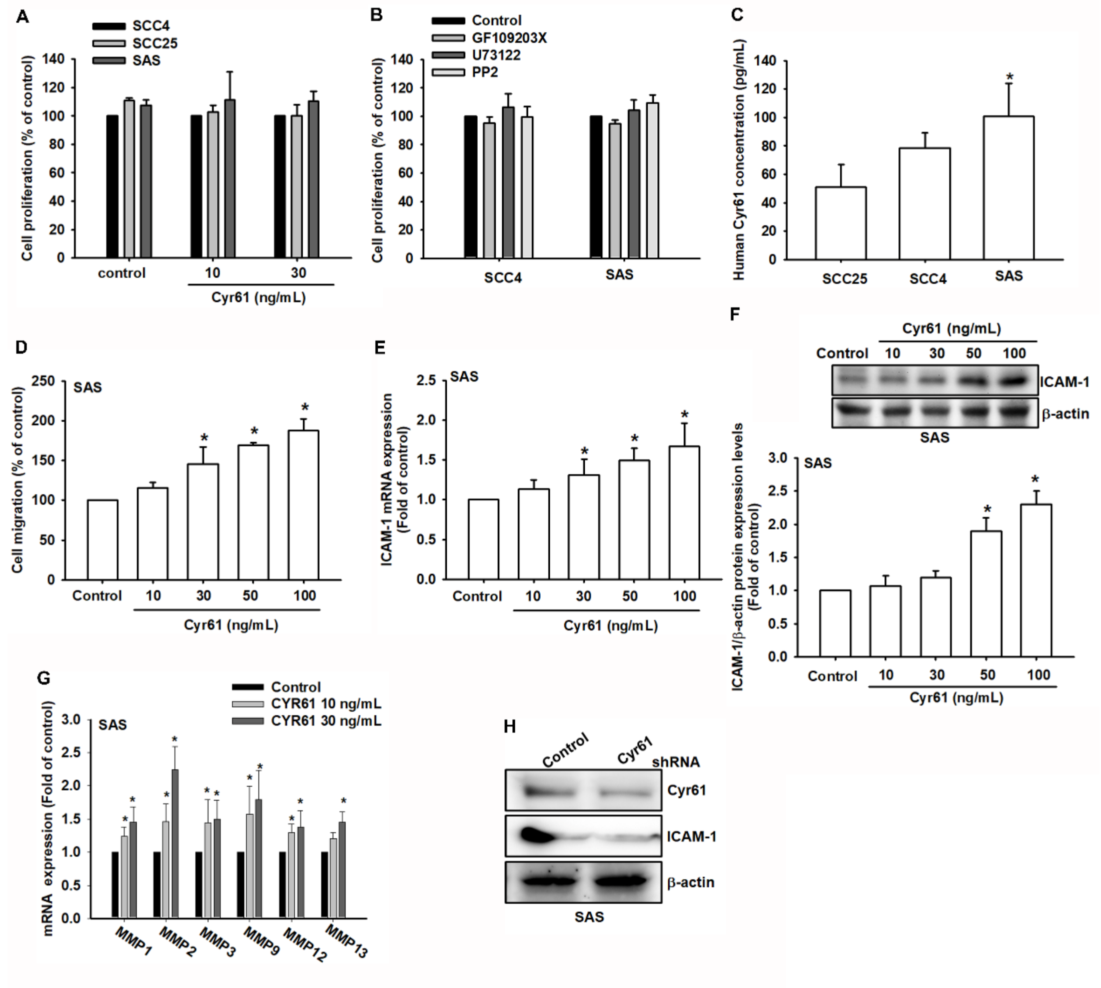

Figure S1. Cyr61 does not affect OSCC cell proliferation but increases migration-related responses and ICAM-1 expression. (A) SCC4, SCC25, and SAS cells were treated with recombinant Cyr61 (0, 10, and 30 ng/mL) for 24 h, and cell proliferation was analyzed by CCK-8 assay (n = 4). (B) SCC4 and SAS cells were pretreated with GF109203X (5 μM), U73122 (1 μM), or PP2 (5 μM) for 24 h, and cell proliferation was analyzed by CCK-8 assay (n = 4). (C) SCC25, SCC4, and SAS cells were cultured in serum-free medium for 24 h, and secreted Cyr61 levels in conditioned medium were measured by ELISA (n = 4). (D) SAS cells were treated with recombinant Cyr61 (0, 10, 30, 50, and 100 ng/mL), and cell migration was analyzed by Transwell migration assay (n = 4). (E) SAS cells were treated with recombinant Cyr61 (0, 10, 30, 50, and 100 ng/mL), and ICAM-1 mRNA expression was analyzed by qPCR (n = 4). (F) SAS

cells were treated with recombinant Cyr61 (0, 10, 30, 50, and 100 ng/mL), and ICAM-1 protein expression was analyzed by Western blot (n = 4). (G) SAS cells were treated with recombinant Cyr61 (0, 10, and 30 ng/mL), and the mRNA expression of MMP1, MMP2, MMP3, MMP9, MMP12, and MMP13 was analyzed by qPCR (n = 4). (H) SAS cells were transfected with control shRNA or Cyr61 shRNA. After 24 h, Cyr61 knockdown efficiency and ICAM-1 protein expression were analyzed by Western blot. Data are presented as mean ± SD of four independent experiments. $*p < 0.05$ compared with the control group.
